# Genetic diversity, essential oil’s chemical constituents of aromatic plant *Mesosphaerum suaveolens* (L.) Kuntze Syn. *Hyptis suaveolens* (L.) Poit. and its uses in crop protection: a review

**DOI:** 10.3389/fpls.2024.1454146

**Published:** 2024-09-19

**Authors:** Armel Frida Dossa, Nicodème V. Fassinou Hotegni, Sognigbé N’Danikou, Eléonore Yayi-Ladekan, Charlotte A. O. Adjé, Latifou Lagnika, Aimé H. Bokonon-Ganta, Enoch G. Achigan-Dako

**Affiliations:** ^1^ Genetics, Biotechnology and Seed Science Unit (GBioS), Laboratory of Plant Production, Physiology and Plant Breeding, Faculty of Agricultural Sciences, University of Abomey-Calavi, Abomey-Calavi, Benin; ^2^ Ecole d’Horticulture et d’Aménagement des Espaces Verts, Université Nationale d’Agriculture, Kétou, Benin; ^3^ World Vegetable Center, East and Southern Africa, Arusha, Tanzania; ^4^ Laboratoire de Pharmacognosie et des Huiles Essentielles (LAPHE), Faculté des Sciences et Techniques, Université d’Abomey-Calavi, Abomey- Calavi, Benin; ^5^ Laboratoire de Biochimie et Substances Naturelles Bioactives (LBSNB), Faculté des Sciences et Techniques, Université d’Abomey-Calavi, Abomey-Calavi, Benin; ^6^ Laboratoire d’Entomologie Agricole (LEAg), Département des Sciences et Techniques de Production Végétale, Université d’Abomey-Calavi, Abomey-Calavi, Benin

**Keywords:** *Mesosphaerum suaveolens*, genetic diversity, essential oil, chemical constituents, pest management, pharmacology, toxicology

## Abstract

This review provides evidence on the genetic diversity, chemical constituents, and ecotoxicology of *Mesosphaerum suaveolens* ‘ essential oil. It emphasizes the agricultural benefits such as crop protection effectiveness of the plant and highlights the existing knowledge gaps and research perspectives to promote its utilization in agriculture. A systematic and extensive review of the literature was done and all pertinent full-text articles and abstracts were analyzed and incorporated into the review. *Mesosphaerum suaveolens* is used traditionally in pharmacology to treat several diseases such as malaria, constipation, stomach problems, and renal inflammation. It also treats cramps, digestive infections, headaches, and skin infections. To date, very few studies have been conducted worldwide about its genetic diversity. These studies highlighted three morphological variants, the blue-flowering, the white-flowering, and the light-purple flowering *M. suaveolens*. Its wide biological actions may be attributed to the numerous groups of chemical constituents in its essential oil including monoterpenes, sesquiterpenes, and diterpenes. Biological studies highlighted evidence of *M. suaveolens* being used as an antifungal, bactericidal, antimicrobial, insecticidal, and repellent plant. The essential oil extracted from *M. suaveolens* showed significant potential for the control of agricultural pests such as *Sitiophilus zeamais*, *Helicoverpa armigera* and *Helminthosporium oryzae*. *M. suaveolens* is commonly used worldwide as a pesticidal plant in healthcare, agriculture, and food preservation. However, there is a lack of studies concerning the toxicity and effectiveness of isolated potent phytotoxic substances, the efficacy screening in the field, the genetic diversity, the essential oil yield, and productivity. Consequently, further studies are required to fill the knowledge gaps.

## 1 Introduction

Agriculture throughout the world is experiencing significant losses caused by pests. Farmers face significant challenges due to pests and diseases (N’Danikou et al., 2014). The impact of climate change on pest-related issues is considerable, as agriculture is highly dependent on weather conditions (Yohannes et al., 2015). To mitigate damages caused by pests, and diseases, farmers in developing countries use high doses of synthetic products such as insecticides, fungicides, and pesticides (Ahouangninou et al., 2012; Zikankuba et al., 2019). The improper application of these synthetic products has resulted in adverse effects including pest resurgence, pesticide resistance, depletion of biodiversity, and pollution of various compartments of the environment (Ishtiaq et al., 2017). To improve food safety there is an increasing exploration into the use of biopesticides and plant-based solutions. These plant-based solutions reduce the pollution and adverse effects caused by synthetic compounds (Ebadollahi et al., 2020). As proof, biopesticides, exhibit target-specificity, are cost-effective, and are environment-friendly. They do not persist in their surroundings (Isman and Machial, 2006). It is believed that these pesticides hold immense promise in terms of pest control and offer a solution in the face of climate change.

Numerous studies were conducted on the pesticidal properties of crops (Diabaté et al., 2014; Fening et al., 2014; Bazongo et al., 2015). Among green pesticides, essential oils (EOs) have earned more attention in recent years due to their high pesticidal potential for controlling insects in greenhouses, granaries (Ebadollahi, 2013), and a broad spectrum of target action on insect pests (Singh and Pandey, 2018). There is evidence that EOs may be less hazardous than synthetic compounds and may break down rapidly in the environment. Essential oils were tested as a substitute for commercial pesticides to preserve cultural assets in an environmentally friendly way. Several mites and harmful insects are sensitive to the essential oils extracted from various plant families (Ebadollahi et al., 2020; Singh and Pandey, 2018; Hernández-Carlos and Gamboa-Angulo, 2019). Essential oils are defined as volatile liquids and aromatic with strong odors obtained through extraction from different plant parts, such as leaves, flowers, roots, seeds, bark, wood, fruits, peel, and whole plant (Carrubba and Catalano, 2009; Almeida-Bezerra et al., 2017). They are complex mixtures of a variety of volatile compounds with low molecular weight, including terpenoids, phenolic components, and other compounds namely aliphatic components that have an interest in pharmacology, healthcare, cosmetic, food, and agricultural industries (Maurya et al., 2021). Essential oils synthetized by plants are known as biotic defense means against pests including insects, fungi, viruses, and herbivores. They also serve as a means for attracting insects during pollination, for suppressing other plants, and regulating water (Sharmeen et al., 2021). The essential oils are extracted through water or hydrodistillation, steam distillation, and cold pressing (peels of citrus fruits) (Mandal et al., 2007; Başer and Buchbauer, 2010; Sharmeen et al., 2021). Among these techniques of extraction, hydrodistillation seems to be the most common method of essential oil extraction from aromatic plants in laboratory as it is considered to be the most ancient, simplest method, easy to implement, and inexpensive (Kumari et al., 2023; Sousa et al., 2022).

The Lamiaceae family is the sixth-largest flowering plant (angiosperm) family, having a worldwide distribution with about 258 genera and 7,193 species (Harley et al., 2004). The species of the Lamiacea family have worldwide economic importance. They are used widely in the world by different cultures in phytotherapy, as a condiment, or, more scarcely as a food (Sedano-Partida et al., 2020). Many constituents isolated from species within the Lamiaceae family have antioxidant, antibacterial, cytotoxic, anti-inflammation, repellent, and insecticidal properties (Joseph et al., 2020). *Mesosphaerum suaveolens* (L.) Kuntze (Lamiaceae) syn *Hyptis suaveolens* L. (Poit.) is an invasive weed species distributed worldwide in tropical regions, near roads, railway lines, nearby areas, open areas, etc. (Sedano-Partida, 2018). The species typically grows in soils with limited nutrients and fertility (Wulff, 1973). The seeds called pignut or chan have been used for the production of refreshing drinks beverages in Mexico and Taiwan (Hsu et al., 2019). Additionally, *M. suaveolens* has always been used by humans for sustenance, animal feed, fuel, and medicinal purposes for a significant period (Oscar et al., 2020). *M. suaveolens* is commonly used to treat various health issues, such as fever in children, stomach, and digestive problems, renal inflammation, injuries, cramps, headaches, and skin infections (Rocha et al., 2009; McNeil et al., 2011; Jeeva et al., 2019). It also possesses anti-inflammation, anti-ulcer, and antiparasitic properties (Jesus et al., 2009; Agbobatinkpo et al., 2018). It can be used as an insect repellent (Limachi et al., 2019). The medicinal and pesticidal potential of *M. suaveolens* may be attributed to the diverse groups of chemical compounds found in its essential oil. *M. suaveolens* is of particular interest due to the volatile oils present in its leaves and stems which contain bioactive compounds possessing insecticidal and repellent properties (Limachi et al., 2019; Aliyu et al., 2022). The main constituents of *M. suaveolens* essential oil are β-phellandrene, cineole, β-caryophyllene, sabinene, and limonene which exhibit variability based on the plant’s ecological areas (Patel, 2017). The chemical content of essential oil varies according to the environmental conditions, the genotype, the time of collection, the season, ecological zones, extraction techniques, plant stages, and preservation methods (Kpadonou Kpoviessi et al., 2012; Joseph et al., 2020). Previous studies have investigated *M. suaveolens* invasive properties, traditional uses, chemical composition, pharmacological effects, and pesticidal properties. Furthermore, most literature reviews have focused on its pharmacology for medical use. On the basis of the various studies carried out on *Mesosphaerum suaveolens*, this review highlights the information on morphological variability in the species, genetic diversity in the species, and toxicological effect on beneficial organisms that was lacking in previous reviews on *M. suaveolens* essential oil (Mishra et al., 2021; Sedano-Partida et al., 2020). It also suggests limitations including the lack of field validation of laboratory findings, the low yield of essential oil extract. Those limitations should be taken into account in the production process of *M. suaveolens*-based pesticides in order to promote its large-scale use in agriculture. It therefore focuses on genetic diversity, which is an integral part of the variability of the chemical composition and oil yield. Studying the genetic and agronomic variability may provide solutions to the limiting factors (e.g. oil yield) for further improvement of the production of *M. suaveolens* essential oil and its use in crop protection. The search for genotypes with higher oil yield and effective against pests could facilitate the development of insecticides.

## 2 Review method and data extraction

This review involved a literature search with the keywords “*Mesosphaerum suaveolens* “ associated with “genetic diversity”, “essential oil”, “biological activity”, “chemical constituents”, “agriculture”, “mechanism of action”, “ecotoxicology”, “dose of action”, “pharmacology”, and “allelopathy” using databases such as PubMed, ScienceDirect, as well as search engines such as Google Scholar and ResearchGate. A hand searching of the literature was also done to collect additional articles. Publications from 1956 up to December 2023 were considered after validation according to search keywords. Consulted documents were written in French, English, or Portuguese. More than 200 articles were collected and were analyzed. Screening included abstract reading and selecting established criteria. Detailed information was extracted from selected articles to elaborate the different sections of this review.

## 3 Botanical, morphological, and ecological description of *Mesosphaerum suaveolens*

*Mesosphaerum suaveolens* is an aromatic herb that is either an annual or perennial plant from the Lamiaceae family. *M. suaveolens* is originally from tropical America and is now widely dispersed as an invasive weed (Ngozi et al., 2014). It grows upright and branches out, and can reach up to 2 m in height. The species called gros Baume in French is known as pignut or bushmint in English and has a distinct scent resembling mint when crushed. It flourishes abundantly in crowded clusters along streets, gardens, and bushes in tropical regions (Ahoton et al., 2010). The entire plant is covered by soft hairs, with a hollow quadrangular stem that has strong furrows and a taproot. The leaves are simple, up to 5 cm long and 4 cm wide, and have a wide range of shapes, from ovate to cordate, with a rounded or heart-shaped base, a pointed tip, and a coarsely toothed margin. They are attached opposite each other on the stem by a short petiole and are hairy on both sides. The petiole can be up to 3 cm long. The hermaphrodite flowers measure approximately 8 mm in length and are arranged in axillary, sessile glomerules surrounded by five blue petals. The fruit, or seed, is a nut that measures about 2 mm in length, featuring a marked polymorphic characteristic at the tips. *M. suaveolens* has two modes of reproduction, both autogamous and allogamous. It propagates via seeds that are disseminated by wind, water, and occasionally animals and humans. The species’ dimorphic seeds, produced in large quantities at a rate of over 2000 per square meter, enable great invasive capability (Raizada, 2006; Barbosa et al., 2013). *M. suaveolens* is found in warm tropical and subtropical regions characterized by high rainfall, but can also thrive in semi-arid environments. Research shows that the seeds exhibit optimal germination temperatures of 25 to 30°C, with better germination rates observed during the day (84%) rather than in the dark (54%) (Wulff, 1973). *M. suaveolens* is located on heavy, moist soils in the Sahelo-Sudan and Sudan-Sahelian regions, with yearly precipitation ranging from 600 to 1200mm. The species prefers open environments and well-drained soil (Aboh, 2008). Like any other invasive species, it is attacked by only a limited number of pests (Afreen et al., 2018) and contains allelochemical compounds (Chatiyanon et al., 2012; Islam and Kato-Noguchi, 2013).

## 4 Traditional uses of *Mesosphaerum suaveolens*

Originally, *M. suaveolens* is known to be used in folk medicine to heal various diseases including malaria, constipation, inflammation of the kidneys, injuries, cramps, digestive infections, headaches, skin infections, respiration problems, gastric ulcers, infections of the uterus (Conti et al., 2011; Jesus et al., 2013; Iqbal et al., 2021). Traditional healers in Benin, Nigeria, Togo, Kenya, Brazil, and India used different plant parts in the form of decoction, infusion, and teas to treat asthma, colds, fever, nausea, and constipation (Raizada, 2006; Almeida-Bezerra et al., 2022). The leaves of *M. suaveolens* contain pharmacologically important volatile metabolites useful for healing. In Sierra Leone, the roots are traditionally decocted as an appetizer, while the leaf extract mixed with lemon juice is ingested to treat stomach aches (Priya, 2015). The burned leaves and stems are turned to ashes and applied to the body over scarifications (Jesus et al., 2013). In several African countries, the leaves of this plant are used to repel mosquitoes (Abagli et al., 2012; Limachi et al., 2019; Abagli and Alavo, 2020). In Kenya, *M. suaveolens* is used to repel mosquitoes when burned inside rooms (Seyoum et al., 2002). Furthermore, the chemical constituents of *M. suaveolens* have been deemed a promising chemical compound having medicinal potential. These constituents possess antifungal, antibacterial, antioxidant, antimicrobial, and anti-HIV properties (Chatterjee and Pakrashi, 1991; Sedano-Partida et al., 2020). The volatile constituents of *M. suaveolens* including ursolic acid, a pentacyclic triterpenoid have shown effectiveness against the SARS-CoV2 virus responsible for the COVID-19 pandemic (Mishra et al., 2021). Pharmacological studies have also demonstrated its ability to combat human pathogens namely *Staphylococcus aureus* (Rosenbach) (Caryophanales: Staphylococcaceae), *Escherichia coli* (T. Escherich) (Enterobacterales: Enterobacteriaceae), *Candida albicans* (Berkhout) (Saccharomycetales: Saccharomycetaceae), etc (Nantitanon et al., 2007; Agban et al., 2013). The essential oils isolated from *M. suaveolens* have shown significant potential for medicinal use and application; they have been reported to possess antimicrobial and antioxidant activities (Nantitanon et al., 2007). In the fight against human breast cancer, the essential oil of *M. suaveolens* exhibited anti-cancer properties on the cell line (Mishra et al., 2021). Recently, the anti-inflammatory effects of *M. suaveolens* leaf oil tested *in vitro* have been reported by Mohanta et al. (2023).

## 5 Genetic diversity in *Mesosphaerum suaveolens*

*Mesosphaerum suaveolens* is an invasive plant that is spreading throughout the world in tropical and subtropical areas; it grows on all continents (Mishra et al., 2021). *M. suaveolens* is autogamous and allogamous (xenogamy, geitonogamy), and polyploidy allows for a wide variety of morphological and chemical characteristics found in nature. In addition, the sexual reproduction of *M. suaveolens* is favored by insect mediation, which leads to genetic diversity within the species (Aluri and Reddi, 1996). Furthermore, numerous biotic and abiotic factors influence the yield and the components of its essential oil. The chemical variation revealed in *M. suaveolens* across various geographical regions has been largely studied and documented by researchers. However, there is very little information on the morphological and genetic diversity of *M. suaveolens* worldwide.

### 5.1 The chromosome number in *Mesosphaerum suaveolens*

Determining the appropriate karyotype is worthwhile for characterizing the genome of a species and for distinguishing closely related species. Thorough knowledge of the distribution of chromosome numbers in angiosperms is fundamental for research in taxonomy, biosystematics, and crop improvement through breeding programs (Coleman, 1982). Chromosome numbers and base numbers were studied for the genus *Hyptis* and reports of variable chromosome numbers within the same species have been documented. For the genus *Hyptis*, polyploid forms are common, with the basic number of chromosomes x = 8, and hybrids can occur (Morton, 1962). Earlier, Morton (1962) reported 2n = 32, whereas the record (Miège, 1960) stands at 2n = 28 for the *M. suaveolens* chromosome number. Vij and Kashyap (1976) somatic studies revealed 2n = 32 in the root-tip cells of *M. suaveolens* with 4x for polyploidy level. This was followed by n = 14 recorded by Bir (1979), 16 chromosomes by Coleman (1982), and 2n = 28 (Krishnappa, 1982). Discordant chromosome numbers 2n = 16, 28, 30, 32, 56, 64 known in *Hyptis* (Darlington and Wylie, 1956; Fedorov, 1969; Moore, 1973) suggested its diabasic nature with x = 7 and 9 (Vij and Kashyap, 1976). A successful breeding program for *M. suaveolens* is needed and requires cytological analysis to remove the ambiguity about the number of chromosomes, and to evaluate the influence of germinal cells on the phenotype.

### 5.2 Morphological and molecular diversity in *Mesosphaerum suaveolens*

**Figure 1:**
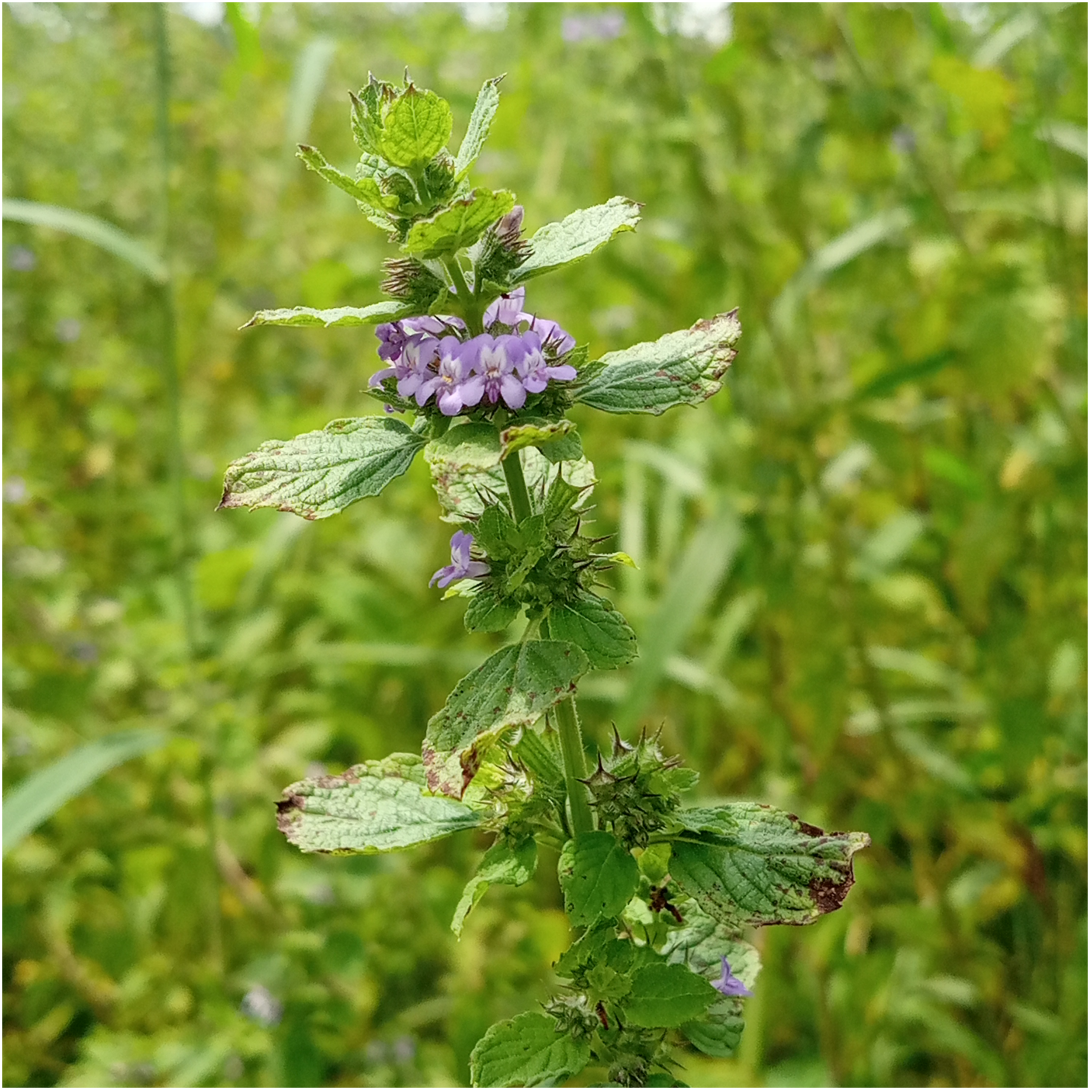
*Mesosphaerum suaveolens* typical form.

**Figure 2:**
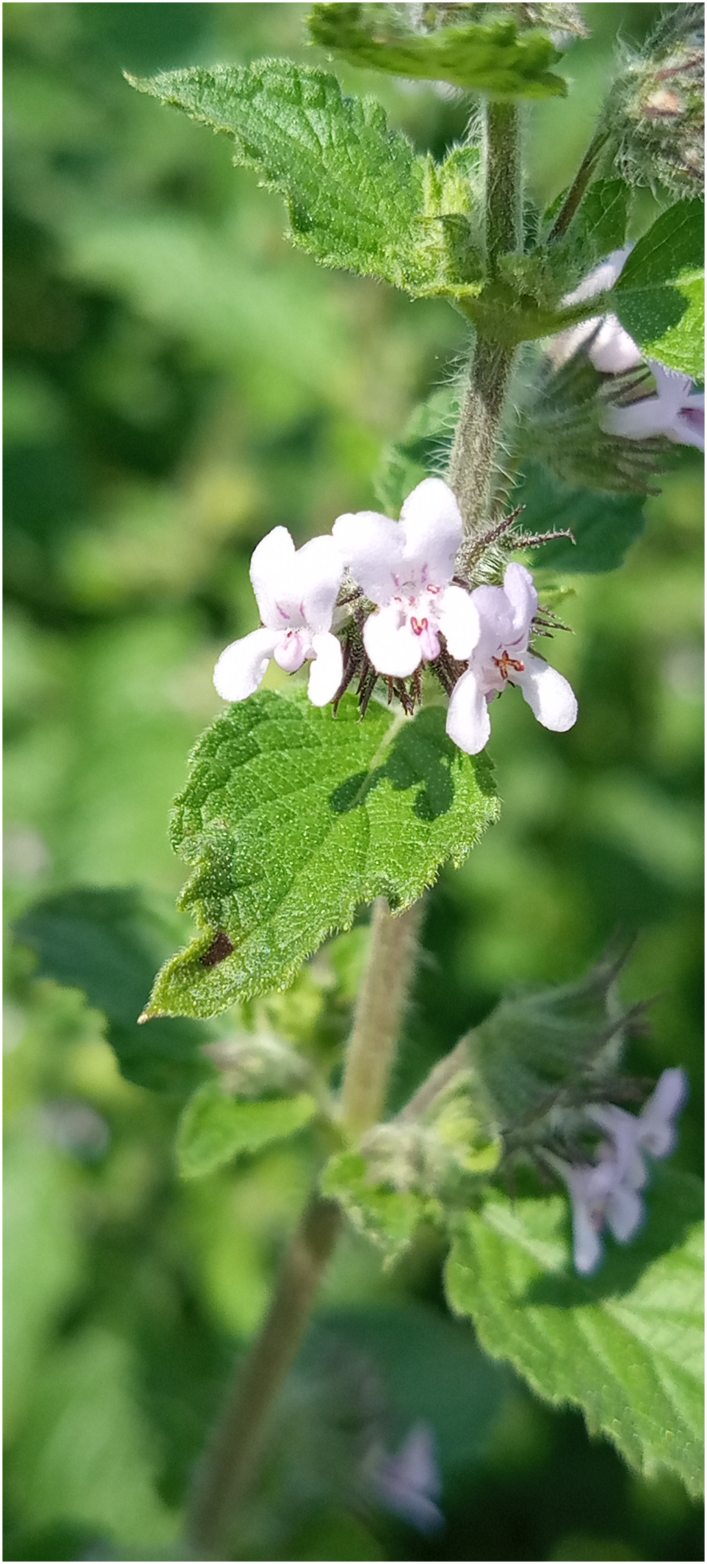
*Mesosphaerum suaveolens* light purple-flowering variant.

For future use in breeding programs, genetic diversity studies in native plants are an essential way of conserving the species and selecting genes and alleles of interest (Enyew et al., 2022). *M. suaveolens* displayed genotypic polymorphism and plasticity at both morphological and physiological levels (Barbosa et al., 2013). *M. suaveolens* has easily distinguishable morphological variation in flower color, leaf size, shape, and petiole length (Mallavarapu et al., 1993; Gadidasu et al., 2011; Johnson et al., 2018). Two morphological variants in the species including the white-flowering and the blue-flowering were reported in India and Brazil by Gadidasu et al. (2011). In Benin, a light-purple-colored flower variant in *M. suaveolens* (**Figures 1**, **2**) was found through empirical observations during fieldwork in the Sudanian phytogeographical region in Gogounou and Boukombé municipalities. The Sudan semi-arid zone has annual rainfall ranging from 900 to 1,100 mm, with a significant rainfall deficit. The average annual temperature is 27.5°C and the relative humidity is 58%. The vegetation consists mainly of tree, woodland and shrub savannah. There are also gallery forests (Akoègninou et al., 2006). Earlier, key observations such as the intra-population variation in seed germination (Wulff, 1973), seed dimorphism, variability in seed size (large and small), seed weight, and chemical variability (Azevedo et al., 2001) were used to explain the genetic diversity within the species (Mandal et al., 2010).

Molecular diversity in *M. suaveolens* has been studied by Gadidasu et al. (2011) using inter-simple sequence repeat (ISSR). The first genetic diversity analysis in the species revealed a 35% polymorphism between white and blue flowering. Inter-simple sequence repeat (ISSR) markers have the advantage of being reproducible at low costs, and do not require prior knowledge of DNA sequences to develop specific primers for the species being analyzed (Coral et al., 2016). However, ISSR markers have their limitations in resolving the geographical differentiation of population in particular when using few genetic markers (Ji et al., 2022). Thus, there is a need to explore further markers with extraordinarily high-throughput genotyping, to assess the extent of genetic variability in *M. suaveolens* worldwide and specifically everywhere *M. suaveolens* chemotypes have been reported to detect the appropriate genotypes in terms of quality and quantity of essential oil.

Obtaining a sufficient quantity of *M. suaveolens* essential oil could be an important factor in facilitating its wide utilization in agriculture. Indeed, the percentage of oil yield from *M. suaveolens* leaves varies from 0.1% to 0.4% by hydrodistillation (Mallavarapu et al., 1993; Tripathi et al., 2009; Johnson et al., 2018; Joseph et al., 2020) which is very low compared to many aromatic plants. It has been reported that many factors affecting the yield and content of essential oil include differences between genotypes, agronomic factors, and the processing and storage of aromatic plant parts (Martins et al., 2006; Silva et al., 2000; Martins et al., 2009; Sałata et al., 2020). However, few scientific publications have addressed the yield component and chemical content of *M. suaveolens* essential oil linked to genotypes. Therefore, research investigations on *M. suaveolens* essential oil could be oriented towards both, the development of high oil-yielding *M. suaveolens* based on the existing diversity, and the establishment and promotion of best agricultural practices.

## 6 Chemical composition of the essential oil of *Mesosphaerum suaveolens*

The assessment of both qualitative and quantitative values of chemical constituents in Lamiaceae species can be very useful to exploit their potential and benefits for agricultural use. The useful biological properties of *M. suaveolens* are attributed to the presence of numerous chemical constituents reported in its essential oil. Knowledge of the main constituents of the essential oil could probably help to predict the type of biological activity for which it might be used for. Monoterpenes and diterpenes have been reported as the main constituents that may induce the phytotoxic properties of *M. suaveolens* (Almeida-Bezerra et al., 2018; Sharma et al., 2019). The compound 1,8-cineole has been identified as the major constituent responsible for the fungistatic and fungitoxic properties (Sharma et al., 2007), and the larvicidal property against *Aedes aegypti* (Linnaeus) (Diptera: Culicidae) (Luz et al., 2020). Moreover, the allelopathic activity found in the essential oil of *M. suaveolens* could be attributed to a synergistic effect of β-caryophyllene (18.6%), sabinene (16%) and spatulenol (11%), the major constituents (Almeida-Bezerra et al., 2018). Chemical content variation has occurred in some species creating different chemotypes with varying amounts of major compounds in their essential oils. The chemical content variation in essential oils composition is influenced by the natural ecosystem in which the aromatic plants grow. This involves the level of soil fertility, the genotype, the relief, photoperiod, irrigation regime, stage of development, etc (Azevedo et al., 2001, 2002; Oliveira et al., 2005; Grassi et al., 2008). There are at least five chemotypes of *M. suaveolens* that have been reported, namely: (1) 1,8-cineole and sabinene, (2) fenchone and limonene, (3) eugenol and germacrene D, (4) β-caryophyllene and 1,8-cineole and (5) sabinene and β-pinene (Pino et al., 2013). The major compounds isolated from *M. suaveolens* plant parts collected around the world are mostly terpenoids including monoterpenes, sesquiterpenes, and diterpenes (Li et al., 2020). They include1,8-cineole, Sabinene, β-pinene, fenchone, α -phellandrene, limonene, eugenol, Linalol, β-caryophyllene, E-caryophyllene, bicyclogermacrene, caryophyllene oxide, germacrene-D, bicyclogermacrene, and terpinolene. Furthermore, the percentage of major elements in essential oil differs from one area to another within the same country, as well as from one continent to another and the plant parts used for oil extraction (**Table 1**). This variability is mainly related to genotypes that are often influenced by geographical distribution. Future assessments of *M. suaveolens* essential oil chemical composition might include the genetic and agromorphological diversity. Therefore, assessing and quantifying the genetic variability among genotypes of *M. suaveolens* for traits such as chemical constituents, and oil yield could help identify pathogen-specific and high oil yielding genotypes. Constituents such as β-caryophyllene and 1,8-cineole are the most common worldwide, with β-caryophyllene almost always reported as the main component. These two compounds have been shown to have biological activities against many pathogens (Sharma et al., 2007; Almeida-Bezerra et al., 2018). Identifying major components in biological activity assays allows the identification of potential bioactive components. However, very few studies were carried out to evaluate the biological activity of the bioactive constituents isolated from *M. suaveolens* essential oil. Therefore, it appears that there are opportunities to use bioactive constituents isolated from *M. suaveolens* essential oil to control several types of insect pests.

**Table 1:** Major compounds in genetic materials of *Mesosphaerum suaveolens*.

Countries	Plant parts	Chemical compound and amounts	References
Australia	Leaves	1, 8-cineole (32%), and β- caryophyllene (29%)	(Peerzada, 1997)
Benin	Leaves	β-caryophyllene (43.7%); trans-α-bergamotene (6.3%); 6-hydroxycarvotanacetone (4.4%); caryophyllene oxide (4.4%)	(Kossouoh et al., 2010)
Benin	Leaves	Terpinen-4-ol (41%), Linalol (14.7%); trans- α -Bergamotene (4.8%)	(Adjou et al., 2019)
	Leaves	1,8-cineole (12%); β-caryophyllene (10.4%); fenchone (11.8%)	(Salifou et al., 2020)
Brazil	Entire plants in vegetative stage	Sabinene (2.93-31.13%), limonene (3.6-17.56%), 1,8-cineole (15.08%), bicyclogermacrene (2.38-12.68%)	(Azevedo et al., 2001).
Brazil	Entire plants in fruiting stage	1,8-cineole (1.08–27.65%) and sabinene (1.22–15.67%), spathulenol (9.25–22.44%), (E)-caryophyllene (0.74–19.7%)	(Azevedo et al., 2002)
Burkina-Faso	Leaves	Sabinene (14%); eucalyptol (12.8%), trans-Oxide β-caryophyllene (11.3%)	(Bayala et al., 2020)
Cuba	Leaves	Caryophyllene oxide (35%)	(Pino et al., 2013)
Nigeria	Immature leaves	Caryophyllene oxide (10.3%); α- phellandrene (10.6%); β-caryophyllene (22.3%)	(Ogunbinu et al., 2009)
Nigeria	Leaves	Caryophyllene (20.6%); sabinene (16.7%)	(Kubmarawa et al., 2015)
Nigeria	Fruits	Oxygenated terpenes (66.8%), 1,8-cineole (29.5%); fenchone (17.2%)	(Essien et al., 2019)
Nigeria	Stems	β-pinene (20.9%); estragole (16.3%); terpenoid hydrocarbons (75.5%)	(Essien et al., 2019)
Nigeria	Leaves	Bicyclogermacrene (8.5%), γ-elemene (8.8%); β-elemene (39.7%)	(Olonisakin et al., 2018)
Nigeria	Stems	β-pinene (20.9%); estragole (16.3%); terpenoid hydrocarbons (75.5%)	(Essien et al., 2019)
Nigeria	Leaves	Bicyclogermacrene (8.5%), γ-elemene (8.8%); β-elemene (39.7%)	(Olonisakin et al., 2018)
Tanzania	Leaves	β-elemene (10.4%); β- caryophyllene (26%)	(Malele et al., 2003)
India	Leaves	1,8-cineole (31.5-35.3%)	(Mallavarapu et al., 1993)
India	Leaves	1,8-cineole (44.4%), β-pinene (11.7%), β-caryophyllene (10%)	(Tripathi et al., 2009)
India	Leaves	α-humulene (9.6%), 5-caranol (18.5%)	(Beena et al. (2008)
India	Leaves	Sabinene (14.7%), allo-aromadendrene (9.3%) and β-caryophyllene (25.7%)	(Vijay et al. (2011)
Indonesia	Leaves	β-caryophyllene (34.65%), germacrene-D (10.3%)	(Chatri et al., 2014)
Italy	Leaves	Terpinolene (10.7%), β-caryophyllene (11.2%), and sabinene (34%)	(Benelli et al., 2012)
Italy	Leaves	Oxygenated sesquiterpenes (2.4%), monoterpene hydrocarbons (64%), oxygenated monoterpenes (8%), and sesquiterpene hydrocarbons (24%)	(Conti et al., 2011)
Ivory-coast	Leaves	β-caryophyllene (33.9%); germacrene-D (25.4%)	(Johnson et al., 2018)
Venezuela	Leaves and flowers	1,8-cineole (19%) and (13.3%), fenchone (18.5%) and (16.1%), bicyclogermacrene (12.7%) and (18.8%)	(Tesch et al., 2015)
Vietnam	Leaves	germacrene-D (11%) and eugenol (68.2%)	(Hac et al., 1996).
Vietnam	Leaves	β-caryophyllene (31%), caryophyllene oxide (17.6%), phytol (9.9%), germacrene-D (6.7%)	(Chung et al., 2020)
Vietnam	Flowers	β-caryophyllene (33.7%), caryophyllene oxide (3.9%), phytol (2.7%), germacrene-D (6.6%)	(Chung et al., 2020)

## 7 *Mesosphaerum suaveolens* essential oil success stories in crops protection

The essential oil of *M. suaveolens* is a biological alternative to synthetic products for pest management in agriculture. The biological properties found in *M. suaveolens* such as cytotoxic, antimicrobial, and insecticidal through its essential oils have shown its value as a source of bioactive compounds (Kuhnt et al., 1995; Abagli and Alavo, 2020). In agriculture, the efficacy of *M. suaveolens* essential oils was proven against various pest categories mainly insects, micro-organisms (fungi and bacteria), nematodes, and weeds (**Tables 2a**–**d**). Its efficacy has been demonstrated to a greater extent in the context of stock pests (stored food pest) than in the field. Indeed, the literature review revealed that no direct utilization of *M. suaveolens* essential oil in the field, nor on field pests, were carried out. Nevertheless, a few isolated direct field trials were conducted using aqueous extracts of *M. suaveolens*. Notable examples include the studies carried out by Biao et al. (2018) and Kossou et al. (2007), which demonstrated the successful control of aphids and thrips, respectively. The efficacy of aqueous extracts of *M. suaveolens* in field settings may serve as a potential indicator of success for the essential oil, which should be taken into consideration when testing the effects of the essential oil in a farming environment. In light of the significance of this element, it is advisable to conduct field trials with a view to corroborating the findings of laboratory studies and also to evaluate the efficacy of the *M. suaveolens* essential oil in combating insects that infest crops in the field and thereby affect yields. *In vitro* and *in vivo* screening should be undertaken throughout the process of identifying and developing *M. suaveolens* biopesticide products.

**Table 2A:** *Mesosphaerum suaveolens* essential oil in insects and acarids control.

Plants parts utilised	Crops	Oil/extracts	Extraction methods	Positive controls	Negative controls	Application modes	Doses applied	Pathogens	Effects observed	Reference
Leaves + branches	*Vigna unguiculata*	Essential oil	Steam distillation (water vapor extraction)	–	Glass vial containing insects without oil	Fumigation: insects released in glass vial full of oil vapor	150 μl	*Callosobruchus maculatus*	(20%) of mortality, (0%) of egg hatch and adult emergence	(Kéıüta et al., 2000)
Fresh leaves	*Zea mays* *Oriza sativa*	Essential oil	Hydrodistillation for 2 h in a Clevenger apparatus.	–	Untreated Petri dishes with essential	Contact toxicity tests in Petri dishes	0.4 μl	*Sitophilus granarius*	(100%) of mortality	(Conti et al., 2011)
Fresh leaves	*Cereal* (grains)	Essential oil	Untreated Petri dishes with essential		Untreated Petri dishes with essential	Repellency tests on filter paper	2 × 10−4 μl/cm^2^	*Sitophilus granarius*	(100%) repellency	(Conti et al., 2011)
Dried Leaves	*Zea mays*	Essential oil	Hydrodistillation in a Clevenger apparatus.	–	Untreated paper disc	Direct contact: Paper disc diffusion method	5 μl. l^-1^	*Sitophilus zeamais’s larvae*	Reduction of the emergence of *Sitiophilus zeamais* larvae by 87%	(Johnson et al., 2018)
Leaves	*Arachis hypogaea*	Essential oil	Hydrodistillation of fresh leaves	–	Untreated insects	Contact toxicity method (insects released on treated seeds)	0.5 µl g^-1^ of peanut	*Tenebroides mauritanicus*	(100%) of mortality	(Adjou et al., 2019)
Leaves	*Arachis hypogaea*	Essential oil	Hydrodistillation of fresh leaves		Paper disc treated with acetone	Repellency tests (paper disc diffusion method)	0.03-0.3/cm^2^	*Tenebroides mauritanicus*	(100%) repellency	(Adjou et al., 2019)
Fresh aerial parts	*Vigna unguiculata*	Essential oil	Hydrodistillation in a Clevenger during 3hours	Cowpea seeds treated with 0.5 ml pure acetone	Untreated Cowpea seeds	Contact toxicity: insects released on seeds mixed with oil in a glass bottle	0.01 ml g^-1^	*Callosobruchus maculatus*	(100%) mortality of adults	(Olonisakin et al., 2018)
Whole plant	*Zea mays*	Essential oil	Steam distillation of the plant powder for 5hours		Untreated	Disc diffusion	0.4 ml	*Sitophilus* *Zeamais*	(100%) mortality of adults	(Abdulmalik et al., 2018)
Leaves	*-*	Essential oil Ct β-caryophyllène (20.69%)	Hydrodistillation	Alphacypermethrin	Tween-80, diluted at 2% in distilled water	Larval immersion test	LC50: 45 mg ml^-1^ LC95: 138.2 mg ml^-1^	*Rhipicephalus (Boophilus) microplus*	(50% and 100%) mortality of larvae after 24h	(Salifou et al., 2020)
Leaves	*-*	Essential oil Ct 1,8-cinéole (Eucalyptol)	Hydrodistillation	Alphacypermethrin	Tween-80, diluted at 2% in distilled water	Larval immersion test	LC50: 45 mg ml^-1^ LC95: 138.2 mg ml^-1^	*Rhipicephalus (Boophilus) microplus*	(50% and 100%) mortality of larvae after 24h	(Salifou et al., 2020)

**Table 2B:** *Mesosphaerum suaveolens* essential oil in plants diseases control.

Plants parts utilised	Crops	Oils	Extraction methods	Positive controls	Negative controls	Application modes	Doses applied	Pathogens	Effects observed	Reference
leaves	*Oryza sativa*	Essential oil	Steam distillation	–	–	Microdilution technique (Contact toxicity)	Inhibitory concentration of 0.4%.	*Helminthosporium oryzae*	Fungitoxicity (Inhibited the growth of fungi)	(Pandey et al., 1982)
leaves	*Brassica caulorupu*	Essential oil	Steam distillation	–	Potato-dextrose agar (PDA) medium + inoculum of the test fungi	Poisoned food technique	100 to 5,000 ppm	*Sclerotinia sclerotium*, *Sclerotium rolfsii* and *Rhizoctonia solan*	(50% to 100%) inhibition of mycelial growth	(Singh and Handique, 1997)
Leaves	*Zea mays*; *Oryza sativa*; *Triticum vulgare*; *Cajanus cajanus*; *Lens esculentum*, *Vigna mungo*; *Arachis hypogea; Anacardium occidentale*;*Cumin cyminum Coriandrum sativum*	Essential oil	Hydro-distillation for 5h	–	Medium treated with distilled water +inocula of the test fungi	Poisoned food technique	MIC500 ppm (fungistatic) ≥1000 ppm (fungicidal)	*Aspergillus flavu*s Link, *Aspergillus niger* Van Tieghem, and *Aspergillus* *ochraceous* Wilhelm	(100%) Inhibition of mycelial growth; (100%) inhibition of spore germination after 10 days of incubation	(Sharma et al., 2007)
	*Zea mays*; *Oryza sativa*; *Triticum vulgare*; *Cajanus cajanus*; *Lens esculentum*, *Vigna mungo*; *Arachis hypogea*; *Anacardium occidentale*;*Cumin cyminum Coriandrum sativum*	Essential oil	Essential oil	–	Petri dish containing water pipetted on to the sterilized cotton swab+disc of inoculum	Volatile activity assay	MIC500 ppm (fungistatic) ≥1000 ppm (fungicidal)	*Aspergillus flavu*s Link, *Aspergillus niger* Van Tieghem, and *Aspergillus* *ochraceous* Wilhelm	(100%) Inhibition of mycelial growth; (100%) inhibition of spore germination after 10 days of incubation	(Sharma et al., 2007)
Leaves	*Gladiolus* spp.	Essential oil	Hydro distillation for 5 h	–	Medium treated with distilled water +inocula of the test fungi	Poisoned food technique (PF) and volatile	0.998 and 1.372 μgml−1	*Fusarium oxysporum* sp. gladioli strain MPPLU 01	-(100%) Inhibition of conidial germination after seven days -Death of fungus after seven days -(100%) Mycelial growth inhibition after seven days	(Tripathi et al., 2009)
	*Gladiolus* spp.	Essential oil	Hydro distillation for 5 h	–	Petri dish containing water pipetted on to the sterilized cotton swab+disc of inoculum	Volatile activity assay (VA)	0.998 and 1.372 μgml−1	*Fusarium oxysporum* sp. gladioli strain MPPLU 01	-(100%) Inhibition of conidial germination after seven days -Death of fungus after seven days -(100%) Mycelial growth inhibition after seven days	(Tripathi et al., 2009)
Leaves	–	Essential oil	Hydrodistillation	–	Growth media without essential oil	Macrodilution in broth and the poisoned substrate technique (dilution in solid medium)	MIC 40 µl/ml MFC 80 µl/ml	*A. parasiticus*, *A. fumigates*, *A. flavus* and *A. niger*	(100%) inhibition of the mycelial growth; decreased conidiation, leakage of cytoplasm, loss of pigmentation and disrupted cell structure suggesting fungal wall degeneration	(Moreira et al., 2010).
Leaves	–	Essential oil	Hydrodistillation of fresh leaves	-Substrate with antifungal drug (ketoconazole)	Substrate without antifungal drug	Macrodilution in broth and the poisoned substrate technique (dilution in solid medium)	1000 μgml−1 1500 μgml−1	*-Fusarium moniliforme* *-Mucor sp*	-growth 25% or less that of the control -No visible growth	(Malele et al., 2003)
Leaves	*Vigna unguiculata*	Essential oil	hydrodistillation	Medium treated with chloramphenicol) 50μg/ml	Medium treated with distilled Water	Modified macro-broth dilution technique was	MIC: 0.5 ml/mm	*Aspergillus niger* *Aspergillus niger Fusarium solani*	-0.83 mm; -0.73 mm; and -1.10 mm growth inhibition	(Olonisakin et al., 2018)

**Table 2C:** *Mesosphaerum suaveolens* essential oil in nematode control.

Plants parts utilised	Crops	Extract	Extraction methods	Positive controls	Negative controls	Application modes	Doses applied	Pathogens	Effects observed	References
Leaves	*Oryzae sativa*	Essential oil	Hydrodistillation	Carbofuran	Distilled water	Exposition by contact	20mg ml^-1^	*Heterodera sacchari*	65.58% mortality after 24h; 100% egg hatch inhibition	(Fabiyi et al., 2015)
Leaves	–	Essential oil	Hydrodistillation	–	–	Larval immersion test	–	*Meloidogyne incognita*	(100%) mortality of larvae after 30mins	(Babu and Sukul, 1990)
Leaves	Solanum lycopersicum	Essential oil	Steam distillation during 4 h.	180 *μ*g ml−1 carbofuran (2,3-dihydro-2,2-dimethyl-1-benzofuran-7-yl Nmethylcarbamate, 98%; Aldrich)	0.01 g ml^−1^ Tween-80^®^	Exposition by contact	1000 *μ*g ml^−1^	*Meloidogyne incognita* second-stage juveniles	(11.5%) mortality after 24h	(Barros et al., 2019)

**Table 2D:** *Mesosphaerum suaveolens* essential oil in weeds control.

Plants parts utilised	Oils	Extraction methods	Positive controls	Negative controls	Application modes	Test crops	Doses applied	Effects observed on crops and weeds	Reference
Leaf	Essential oil	Hydrodistillation in Clevenger apparatus 150 g/2L of distilled water for 2h	–	Untreated seeds	Direct contact in petri dishes (immersion)	*Pilosocereus gounellei* subsp. Gounellei (seeds)	1000 μg ml^-1^	(59,75%) inhibition of the seeds germination	(Almeida-Bezerra et al., 2020)
Dried leaves	Essential oil	Hydrodistillation system, 200g/4 L of distilled water boiled for 2 h	–	Untreated seeds	Direct contact in petri dishes (immersion)	*Cereus jamacaru DC.* subsp. jamacaru (seeds)	1000, 500, and 250 μg ml^-1^	(100%) inhibition of the seeds germination over 7days	(Almeida-Bezerra et al., 2018)
Fresh leaves	Fresh leaves	Hydrodistillation system, 300kg in distilled water boiled for 3 h	–	Tween-20 (surfactant) in distilled water	Direct contact in petri dishes containing filter paper moistened with Essential oil	*Echinochloa crus-galli* (seeds)	2 mg ml^-1^	(100%) germination and early growth inhibition	(Sharma et al., 2019)
Fresh leaves	Fresh leaves	Hydrodistillation system, 300kg in distilled water boiled for 3 h	**-**	Tween-20 (surfactant) in distilled water	Direct contact in petri dishes containing filter paper moistened with Essential oil	*Oryza sativa* (seeds)	2 mg ml^-1^	(40%) germination and early growth inhibition	(Sharma et al., 2019)

### 7.1 *Mesosphaerum suaveolens* essential oil in the control of insects and acarids

The research studies on the evaluation of the insecticidal properties of *M. suaveolens* point to the use of the essential oils of *M. suaveolens* (**Table 2a**). Its essential oil has been used as a biopesticide to protect cowpea, rice, maize, stored cereals, groundnut kernels, cashew nut, spices cumin, coriander, horticultural crops, and peanut against numerous insects such as *Sitophilus zeamais* (Motschulsky) (Coleoptera: Curculionidae), *Tenebroides mauritanicus* (Linnaeus) (Coleoptera: Trogossitidae), *Callosobruchus maculatus* (Fabricius) (Coleoptera: Bruchidae), *Megalurothrips sjostedti* (Trybom) (Thysanoptera: Thripidae); *Aphis craccivora* Koch (Hemiptera: Aphididae), and *Maruca vitrata* (Fabricius) (Lepidoptera: Crambidae) (Kéïta et al., 2000; Tripathi and Upadhyay, 2009; Johnson et al., 2018; Adjou et al., 2019). The essential oils of *M. suaveolens* used in different combinations or alone have efficiently controlled different pests by direct contact, ingestion, or systemic toxicity through repellent, fumigants, larvicide, adulticide, or growth inhibiting activity (Mishra et al., 2021). Essential oil vapors (by fumigation) of *M. suaveolens* leaves caused no eggs hatching and no adults emergence even after 30 days while it caused low mortality (< 20%) of adult *C. maculatus* 24 h after during storage in a glass vial at a concentration of 150 µl (Kéïta et al., 2000). Similarly, the leaf essential oil reduced the emergence of treaded *S. zeamais* larvae by 87% at a concentration of 5 μl l-^1^ (Johnson et al., 2018). The major constituents found in the essential oil that may be responsible for the insecticidal properties were Germacrene D (25.4%) and β-Caryophyllene (33.9%) (Johnson et al., 2018). At a concentration of 0.5 µl essential oil/g groundnut, 100% mortality of *T. mauritanicus* was observed after 24 h; the main constituents of this essential oil were linalool (15%), and terpinene-4-ol (41%) (Adjou et al., 2019). The insecticidal activity of *M. suaveolens* essential oil on *Tribolium castaneum* (Herbst) (Coleoptera: Tenebrionidae), *Rhyzopertha dominica* (Fabricius) (Coleoptera: Bostrichidae) *C. maculatus* and *Sitophilus oryzae* (Linnaeus) (Coleoptera: Curculionidae) is ensured by altering the octopamine receptor (Tripathi and Upadhyay, 2009). The presence of these terpenes, as previously mentioned, makes *M. suaveolens* essential oil highly insecticidal, as evidenced by *in vitro* experimentations. However, this remains to be demonstrated *in vivo*. The potential major compounds having insecticidal varied by assay. The insecticidal and repellency activities of *M. suaveolens* essential oil were concentration-dependent. Furthermore, great care must be taken with the method of evaluation, as insecticidal efficacy varies depending on the technique used, as also highlighted by Olonisakin et al. (2018), who recorded 70% mortality for the filer-paper method compared to 100% for the anti-feedant test. From this study, we suggest future research to better understand the mechanisms of action of *M. suaveolens* essential oil in insects to determine how insecticide products can be used in agriculture.

Limited research on the acaricidal properties of *M. suaveolens* essential oil were carried out on polyphage acarids. Salifou et al. (2020) investigated the acaricide activity of two chemotypes of *M. suaveolens* leaves essential oil from Benin against *Rhipicephalus* (*Boophilus*) *microplus* (Canestrini) larvae. The larval mortality observed 24 h after immersion in the oils at a concentration of 0.16%, 0.6%, 1.2%, 2.5%, and 5% w/v was 1.6–66.5% and 90.7- 99.2% for chemotype 1,8-cineole and chemotype β-caryophyllene respectively. The chemotype β- caryophyllene was found to be the most promising candidate for the formulation of bio-acaricides against *Rhipicephalus* (*Boophilus*) *microplus* (Salifou et al., 2020). The present study showing the acaricidal effect of the essential oil of *M. suaveolens* on this cattle acarid is an interesting result that demonstrates the potential of the essential oil of *M. suaveolens* for use in the control of acarids that cause enormous damage to crops such as manioc and tomatoes, both in the field and in the greenhouse. It should also be noted that *M. suaveolens* has been used much more extensively to control acarids on animal and in its aqueous form than in its oil form to control mites (Ohimain et al., 2015). This could be explained by the ease of obtaining the aqueous extract compared to the oil, which requires more suitable equipment. It could also be explained by the quantity of extract aqueous obtained after maceration or grinding, always compared to the small quantity of oil obtained by hydrodistillation.

### 7.2 *Mesosphaerum suaveolens* essential oil in the control of plant diseases

The inhibitory activity at a concentration of 0.4% of *M. suaveolens* essential oil on *Helminthosporium oryzae*, which causes leaf spot disease in rice, was previously reported by Pandey et al. (1982) via invo trials (**Table 2b**). *M. suaveolens* oil inhibited the growth of *Sclerotinia sclerotium*, *Sclerotium rolfsii*, and *Rhizoctonia solani*, soil-borne fungi by half and completely at 100 ppm, and 5,000 ppm respectively. The pathogens were isolated from *Brassica caulorapa*, O*ryza sativa*, and *Cajanus cajan* infected plants. The dose has influenced the biological activity of the essential oil. To see if *M. suaveolens* oil in combination with *Trichoderma harzianum* enhanced the efficacy of the treatment, the combination of *T. harzianum* and oil (10 mg.kg^-1^ + 0.5%) was done and found to be more effective in controlling wilt and rof diseases, achieving a rate of 72%. In comparison, the sole use of *T. harzianum* resulted in 67% disease control (Singh and Handique, 1997). In post-harvest management, Sharma et al. (2007) demonstrated the antifungal activity of *M. suaveolens* leaf oil against storage mycoflora. The oil showed 100% inhibition of mycelial growth of *Aspergillus flavu*s, *Aspergillus niger*, and *Aspergillus ochraceous* at 500 ppm and 1,000 ppm concentration after ten days. The pathogens were isolated from the seeds of *Zea mays*, *Oryza sativa*, *Triticum vulgare*, and *Cajanus cajanus*. Furthermore, *M. suaveolens* leaf essential oil has well inhibited (100%) the mycelial growth of *Fusarium oxysporum* f sp. gladioli in poisoned food technique (PF) and volatile activity assay (VA) respectively. It was also found to be fungicidal at 1.25 and 0.99 µg ml^-1^ concentration of oil in PF and VA, respectively after seven days of incubation (Tripathi et al., 2009). *Mesosphaerum suaveolens* leaf essential oil has shown anti-aspergillus efficacy with minimum inhibitory concentration and minimum fungicidal concentration of 40 and 80 µl ml^-1^, respectively against *A. parasiticus*, *A. fumigates*, *A. flavus*, and *A. niger* (Moreira et al., 2010). In Tanzania, the growth inhibition of *Fusarium moniliforme* fungi was 25% or less than the control while no visible growth was observed for *Mucor* sp. when treated with *M. suaveolens* leaf essential oil at 1000 μg ml^-1^ and 1500 μg ml^-1^ concentration (Malele et al., 2003). This study, for which the commodity was not revealed by the authors, demonstrates varying effects of the essential oil on different pathogens, indicating a pathogen-specific inhibitory effect.

The inhibitory and anti-growth effects of the essential oil of *M. suaveolens* have been demonstrated on a wide range of pathogens and on several cultures of interest, thanks to its variable monoterpene composition. The essential oil was effective at both low and high doses using different evaluation methods (Tripathi et al., 2009; Moreira et al., 2010). However, the fungicidal properties of the oil against these pathogens were mainly demonstrated in the laboratory and greenhouse. Validation of these results in a field environment is an vital needed information that could accelerate the scale-up and use of oils to control plant diseases.

### 7.3 *Mesosphaerum suaveolens* essential oil in the control of nematodes

Limited research on the nematicidal properties of *M. suaveolens* essential oil has been carried out on polyphage nematodes (**Table 2c**). The essential oil was tested on *Heterodera sacchari* eggs and second-stage juveniles at a concentration of 10 mg ml^-1^, 15 mg ml^-1^, and 20 mg ml^-1^. The population of *Heterodera sacchari* used in this *in-vitro* assay was collected on rice cultivar NERICA 1. The biological activity (mortality and inhibition of egg hatching) was highest (65.85%) at a concentration of 20 mg ml^-1^ with beta-caryophyllene and sabinen as the major constituents (Fabiyi et al., 2015). Babu and Sukul (1990), however, reported 100% mortality of *Meloidogyne incognita* larvae 30 minutes after applying essential oils of *M. suaveolens* with major constituents such as D-limonene and menthol. Despite the efficacy mentioned above of *M. suaveolens* against nematodes, *M. suaveolens* essential oils from Brazilian plants did not show conspicuous nematicidal activity (11.5% dead) against *Meloidogyne incognita* as the positive control, carbofuran (58.3% dead) at a concentration of 1000 *μ*g ml^-1^ (Barros et al., 2019). This great difference between the results of Babu and Sukul (1990) and Barros et al. (2019) on *M. incognita* could be explained by the difference in the constituents of the essential oils, as the oils used were extracted from plants from different countries, different environments. This is showing how the environment could affect the biological activity of *M suaveolen*s. From these various study reports, it appears that the effect of essential oil on nematodes varies depending on the concentration of essential oil, the time after application, the chemical composition of the oil, and the pathogen. Despite the success of the laboratory and greenhouse trials, direct testing of the essential oil in the field has not yet been carried out. Field trials would therefore be useful to better understand the effects and, in particular, to determine the required doses.

### 7.4 *Mesosphaerum suaveolens* essential oil in weeds control

Modern agriculture tends to promote the use of herbicides in controlling weeds, which considerably reduces both the quality and quantity of agricultural crop yields by competing with cultivated crops at the soil, space, and light levels (Mehdizadeh and Mushtaq, 2020). The herbicidal property of essential oils (EOs) is considered one of the most valuable ways to control weeds in ecological agriculture (Batish et al., 2008). It is very difficult for certain plant species to thrive near *M. suaveolens* in the natural environment, therefore phytotoxic and cytotoxic properties of *M. suaveolens* EOs have been evaluated in studies to elucidate its activity. Earlier studies started by evaluating the phytotoxicity of EOs on the germination, and seedling growth of various crops and weeds (Chatiyanon et al., 2012; Rodrigues et al., 2012; Islam and Kato-Noguchi, 2013). The essential oil of *M. suaveolens* has been shown to inhibit completely the seed germination of *Cereus jamacaru.* DC. Subsp. Jamacaru (Cactaceae). At a concentration of 1000 μg ml^-1^, 100% inhibition of seed germination of the test species was observed while 86% germination was observed for the control (Almeida-Bezerra et al., 2018). In addition, the terpenes found in the leaves of *Mesosphaerum suaveolens* (L.) Kuntze (Lamiaceae) have also induced a negative allelopathic effect (51% of germination) on the seeds of *Pilosocereu gounellei* (F.A.C.Weber) Byles & Rowley subsp. gounellei at a concentration of 1000 μg ml^-1^ (Almeida-Bezerra et al., 2020). Sharma et al. (2019) evaluated the allelopathic properties of *M. suaveolens* leaves EOs against *Oriza sativa* L. (Poaceae) and its principal weed, *Echinochloa. crus-galli* (L.) P.Beauv. (Poaceae). *M. suaveolens* leaves EOs (≥ 2 mg ml^-1^) had complete (100%) growth inhibitory activity on germination and seedling growth of *E. crus-galli* while (40%) inhibition was observed on *O. sativa*. The crop was less affected than the weed as the inhibition activity was much less on the seed and seedling growth. The authors concluded that *M. suaveolens* essential oil could be used as a bioherbicide in sustainable agriculture. Despite the success of the laboratory and greenhouse trials, direct testing of allelopathic properties of essential oil in the field has not yet been carried out. Field trials would therefore be useful to corroborate greenhouse results.

### 7.5 Mode and mechanism of action of *Mesosphaerum suaveolens* essential oil

Essential oils have several biological activities. The mode of action of essential oils varies depending to the targets, the chemical composition, the entry point and other factors. In general, essential oils could be ingested, inhaled or skin absorbed by pests (Ozols and Bicevskis, 1979; Devrnja et al., 2022). Most monoterpenes act as chemical messengers for animal tissues, insects and plants, disrupting their cell membranes (Isman and Machial, 2006). *Mesosphaerum suaveolens* is rich in monoterpenes and sesquiterpenes and its essential oil has a wide spectrum of antimicrobial activity (Almeida-Bezerra et al., 2022). Very few studies have been conducted on the mechanism of action of *M. Suaveolens* and its main components, the monoterpenes. Tripathi and Upadhyay (2009) suggested that the insecticidal action of *M. suaveolens* essential oil on *Tribolium castaneum, Rhyzopertha dominica*, *Callosobruchus maculatus*, and *Sitophilus oryzae* is ensured by octopamine receptor alteration. Monoterpenes act by penetrating insects body through the cuticle, digestive system and the respiratory system (Gnankiné and Bassolé, 2017). Furthermore, treatment with *M. suaveolens* essential oil-induced inhibitory effect on the growth and morphogenesis of the fungus *F. oxysporum* as well as inhibition of conidial germination, vegetative hyphae damage, and alterations (Tripathi et al., 2009). It is recognized for numerous *M. suaveolens* essential oil constituents a synergy of action in controlling fungus that finally leads to several negative impacts on the cell, basically the lack of cytoplasm, damage of integrity, and ultimately mycelial death (Tripathi et al., 2009). However, only a few studies elucidated *M. suaveolens* cytotoxicity. The first cytotoxicity study was done with *M. suaveolens* leaf extract in 2016 which was found to be cytotoxic on the meristematic root tips of *Allium cepa*, the abnormalities types were non-clastogenic and clastogenic (Sumitha and Thoppil, 2015). The second study done by Sharma et al. (2019) revealed that *M. suaveolens* essential oil induced several mechanisms that involved visible injury, reduction in chlorophyll content, and cell viability leading to total wilting of the plants. Another modes of action are the cell division alteration in the meristematic cells, aberrations at chromosomal and cytological level (Sharma et al., 2019). Regarding the mechanism, the essential oil of *M. suaveolens* exerted anti-proliferative, cytotoxic activity on cancer cell lines by arresting the cell cycle and decreasing the phase (in HeLa cells) (Bayala et al., 2020). From these research results, different mechanisms of *M. suaveolens* are time and dose-dependent. The investment in the discovery of the mode of action of *M. suaveolens* bioactive constituents could be a great step to accelerate pesticide research and development because it could reduce the required time and costs (Pino et al., 2013).

## 8 Effects of *Mesosphaerum suaveolens* essential oil on the environment and ecosystems

### 8.1 Environmental effects of *Mesosphaerum suaveolens*

*Mesosphaerum suaveolens* is an invasive plant that grows quickly and occupies space quickly, adapting to any type of soil, even the most impoverished (Raizada, 2006). The invasive effect of *M. suaveolens* has been proven by many researchers on both weeds and plants of interest for human nutrition and the environment (Almeida-Bezerra et al., 2018, 2020; Sharma et al., 2019). It has been reported that in the Vindhyan dry deciduous forest of India, the local species decrease with increasing *M. suaveolens* population (Sharma et al., 2009). This study also showed that the species composition changed in the *M. suaveolens* dominated locations. Almeida-Bezerra et al. (2020) have shown that the decomposition of *M. suaveolens* leaves releasing the allelopathic compounds could explain the depletion of the weeds of ecological interest, *C. jamacaru* subsp. *jamacaru* and *P. gounellei* subsp. *gounellei*. According to the same authors, this allelopathic effect is due to the presence of triterpenes resulting from the decomposition of the leaves. Moreover, the ecological incidence of *M. suaveolens* evaluated in northwestern India showed a serious reduction in diversity, dominance, richness, and evenness of natural species in the invaded regions compared to the uninvaded regions (Sharma et al., 2017). Numerous economic species found in the uninvaded regions, such as *Paspalidium flavidum* (Retz.) A.Camus. (Poaceae), *Justicia adhatoda* (L.) (Acanthacea), *Carissa carandas* (L.) (Apocynaceae), *Anisomeles indica* (L.) Kuntze (Lamiaceae), *Dioscorea deltoidea* (wall.) (Dioscoreaceae), and *Murraya koenigii* (L.) (Rutaceae) were visibly absent in the invaded regions. In addition to negatively affecting the natural floristic diversity, the invasion of *M. suaveolens* affects the environment by modifying the pH of the soil and nitrogen mineralization processes (Sharma et al., 2017; Afreen et al., 2018). Furthermore, *M. suaveolens* has an indirect effect on the animals of interest (transhumant cattle, sheep, and goats) through the invasion of agro-pastoral ecosystems which leads to the disappearance of natural pastures (Aboh, 2008). These aforementioned effects are due to the permanent presence of *M. suaveolens* within the flora and fauna communities (forest). Used in a fixed-term agricultural space, the effect of *M. suaveolens* on the environment could probably be less given the short duration of effect recognized for these different forms and, above all the volatility of essential oils. The use of *M. suaveolens* allelopathy properties in agriculture might be with caution or even much more oriented towards extracts to preserve diversity. The use of *M. suaveolens* extract forms in agriculture will avoid the contamination of communities by the obvious dispersal of seeds when using the plant in association.

### 8.2 Toxicological effect on beneficial organisms

Most essential oils are non-toxic to warm-blooded animals and are considered “safe” by the Environmental Protection Agency and the Food and Drug Administration in the USA (Ebadollahi et al., 2020). However, in addition to their pharmacological and insecticidal potential, the direct and indirect effects of phytoproducts on non-target organisms such as honey, bees, and natural enemies, as well as economic aspects, must be considered before commercialization (Ebadollahi et al., 2020). To date, only a limited number of studies have evaluated the influences of essential oil treatments on natural biological control agents. This aspect is of great importance to avoid resurgence effects. Crushed leaves and essential oils of *M. suaveolens* repelled naive females of *Dinarmus Basalis* Rondani (Hymenoptera: Pteromalidae) (Sanon et al., 2011). This olfactometer study demonstrated the habituation process to render natural enemies familiar to the biopesticide. The essential oil of *M. suaveolens* was toxic with LC50 of 49.72 and 15.5 μg ml^-1^ in *Artermia salina* (L.) (Anostraca: Artemiidae) and *Drosophila melanogaster* Meigen (Diptera: Drosophilidae) respectively, in contrast to the leaf infusion which was non-toxic to the organisms at all the concentrations tested (Almeida-Bezerra et al., 2017). This study indicated that toxicity of *M. suaveolens* essential oil depends on the dose and frequency of application. The essential oils of *M. suaveolens* were reported to have a moderate toxicity to non-target organisms such as *Danio rerio* (F. Hamilton) (Cypriniformes: Cyprinidae) and *A. salina* at high concentrations (> 500 μg ml^-1^) with 100% survival at lower concentrations. In these ecotoxicological tests, the essential oils were more toxic to *A. aegypti* larvae, the pathogen than to non-target organisms (*D. rerio* and *A. salina*) showing that this essential oil could be safe (Luz et al., 2020). According to Tripathi and Upadhyay (2009), the oil had low persistence, however, many pieces of researches should focus on the toxicological evaluation of *M. suaveolens* essential to preserve the natural existing fauna that contributes to keeping the pest population low.

## 9 Knowledge gaps and research perspectives

The desire to use plant-derived products in pest management has increased worldwide in recent years due to the adverse effects of synthetic pesticides. As a result, many action research projects for the development of biopesticides are being carried out with government support in the context of developing policies for better pest biocontrol (Devrnja et al., 2022). The renewed interest in *M. suaveolens* -derived constituents for pest control is certainly due to the presence of effective toxicological and pharmacological properties noted by scientists (Mishra et al., 2021). Although *M. suaveolens* is among the most studied species of *Hyptis* genus (Barbosa et al., 2013), there is a lot of remaining research questions about allelopathic and pesticidal effects. However, the complexity of the isolation and identification of bioactive molecules, the time for isolation purification bioactivity assays, the perceived high cost, the low oil yield, are some of the main constraints for the discovery process of biopesticides. Another important nodus, different research frameworks, different units of measurement, and different methods, were used to conduct *M. suaveolens* pesticidal research works, and therefore it is quite difficult to compare the essential oil efficacy as a potent plant-derived pesticide. In most cases, the tests were carried out without a positive control; however, some bioactive aqueous extracts were tested for their pesticidal activity on pest population growth, and they were less active than commercial pesticides (Biao et al., 2018; Bonilla-Landa et al., 2022). In contrast, the combination mycorrhizae-extracts of *M. suaveolens* was as effective as a commercial pesticide (Abakar et al., 2020). Some particular limitations that need to be overcome to facilitate the agricultural use of the essential oil of *M. suaveolens* or its isolated constituents are highlighted in the following paragraphs.

- The chemical composition variability of the essential oil of *M. suaveolens* with season, environment, and level of soil fertility, the genotype, the relief, photoperiod, irrigation regime, plant parts, and the development stage. This variability is both a positive (wide range of action), and a limiting factor for standardization. Five chemotypes of the essential oil have been reported for example (Azevedo et al., 2001; Pino et al., 2013).

- The volatility and limited persistence of the essential oil of *M. suaveolens*. The main constraint limiting the use of these biopesticides in agriculture is related to the volatility and limited persistence of essential oil under field conditions (Devrnja et al., 2022).

**-** Variation in oil content (yield) of *M. suaveolens*. For example, the percentage of oil yield from *M. suaveolens* leaves varies between 0.1% and 0.46% when hydrodistilled (Tripathi et al., 2009; Mallavarapu et al., 1993; Johnson et al., 2018; Joseph et al., 2020). Luz et al. (2020) discovered that the yield of essential oil is influenced by the season. Numerous other factors impact the yield and content of essential oils, such as variations amongst genotypes, agronomic factors, as well as the handling and preservation of aromatic plant parts (Sałata et al., 2020). The small leaves and inflorescence yielded the highest percentage of oil at the flowering stage (Sharma et al., 2007).

- The essential oil of *M. suaveolens* displays a high-toxicity level to beneficial animals, such as *Danio rerio*, *Artemia salina*, and *Drosophila melanogaster* at high concentrations and moderate toxicity at lower concentrations (Luz et al., 2020). In addition, the chemical constituents of *M. suaveolens* exhibit toxicity to ecological plant species such as *P. flavidum*, *J. adhatoda* for example (Sharma et al., 2017; Afreen et al., 2018).

- The phytotoxicity of *M. suaveolens* is non-specific and affects numerous crops such as cereals and vegetables of economic interest, including *Lactuca sativa* (L.) (Asteraceae), *Brassica napus* (L.) (Brassicaceae), *Sorghum vulgare* (Moench) (Poaceae) and *Raphanus sativus* (L.)(Brassicaceae) (Chatiyanon et al., 2012; Rodrigues et al., 2012; Islam and Kato-Noguchi, 2013). *Oriza sativa* and *Triticum aestivum* (L.) (Poaceae) were also found to be affected by this phytotoxicity (Poornima et al., 2015). This general phytotoxicity could limit its use in agriculture. However, some crops were found to be less affected (tolerant) than others. For example, *O. sativa* exhibited lower susceptibility than its major weed, E. *crus-galli* (Sharma et al., 2019). This characteristic of the oil renders it valuable for weed control as the essential oils are capable of biodegradation and fall within the GRAS (Generally Regarded as Safe) compounds category (Batish et al., 2008).

- The toxicity of the essential oil of *M. suaveolens* can be influenced by various factors including pathogens, plant species, concentration, and plant part used for extraction. According to Malele et al. (2003), *M. suaveolens* leaves essential oil caused complete growth inhibition on *Mucor* sp. while it resulted in only 25% or less growth inhibition on *Fusarium moniliforme*. The leaves of *M. suaveolens* were the most toxic to the test fungi (Sharma et al., 2007). Moreover, the aqueous leaf extract of *M. suaveolens* demonstrated efficacy against nematodes, whereas the essential oil did not possess nematicidal properties against *Meloidogyne* spp. infecting *Allium cepa* for example (Barros et al., 2019; Okechalu et al., 2020).

- Most of the pesticidal and allelochemical tests for the essential oil of *M. suaveolens* were carried out in laboratory settings. There have been a limited number of studies evaluating the efficacy of *M. suaveolens* essential oils in greenhouses, farming environments, or natural conditions. The limited field trials have focused on assessing the repellent effect on the association between crops and *M. suaveolens* (Kossou et al., 2007), and incorporating leaves and stems as organic amendments for controlling nematodes (Onyeke et al., 2014). The quantity of essential oil required may limit the scope of field trials as *M. suaveolens* has low oil yields.

- The major compounds isolated from *M. suaveolens* are mostly terpenoids including monoterpenes, sesquiterpenes, and diterpenes (Li et al., 2020). *M. suaveolens* has been reported richer in monoterpenes such as 1, 8-cineole, and β-pinene. The monoterpenes were found to be responsible for allelopathy and pesticide properties of *M. suaveolens*. Nevertheless, compound 1, 8-cineole was found as the major constituent of the essential oil that has shown moderate toxicity to *Artemia salina* and *Danio rerio* (Luz et al., 2020). Similarly, the essential oil of *M. suaveolens* having sabinene, and β-Caryophyllene as major constituents has exhibited toxicity to *Artermia salina* and *Drosophila melanogaster* (Almeida-Bezerra et al., 2017).

If the aforementioned limitations can be efficiently addressed, there is tremendous potential for using pesticidal and allelopathic properties of *M. suaveolens* essential oil in sustainable agriculture.

To further facilitate the potential utilizations, futures researches should focus on the following actions.

1- Validating the pesticidal and allelopathic activities at the field level to better assess and take into account the environmental impact on the efficacy and effectiveness of *M. suaveolens* essential oil and its isolated constituents.

2- Exploring the toxicological effect of *M. suaveolens* essential oil on insects and their natural enemies, with other beneficial organisms for effective and efficient application.

3- Investigating the phytotoxicity of *M. suaveolens* essential oil and its isolated constituents on diverse crops and weeds using the hormetic dose-response model. The determination of application rates that result in hormetic doses of essential oil and its isolated constituents in the early stages of the crops may also contribute to the suppression of weed growth by promoting crop growth (Islam and Widhalm, 2020).

4- Developing a list of the most sensitive crops, and the most sensitive plants of ecological interest to the essential oil, and isolated constituents. It is therefore important to know which plants are not affected by the allelopathic activity of *M. suaveolens* in terms of yield, seed emergence, growth, and development, so that *M. suaveolens* can be cultivated in nearby areas and also used as an essential oil to control their predators (weeds, insects, viruses etc.).

**Figure 3:**
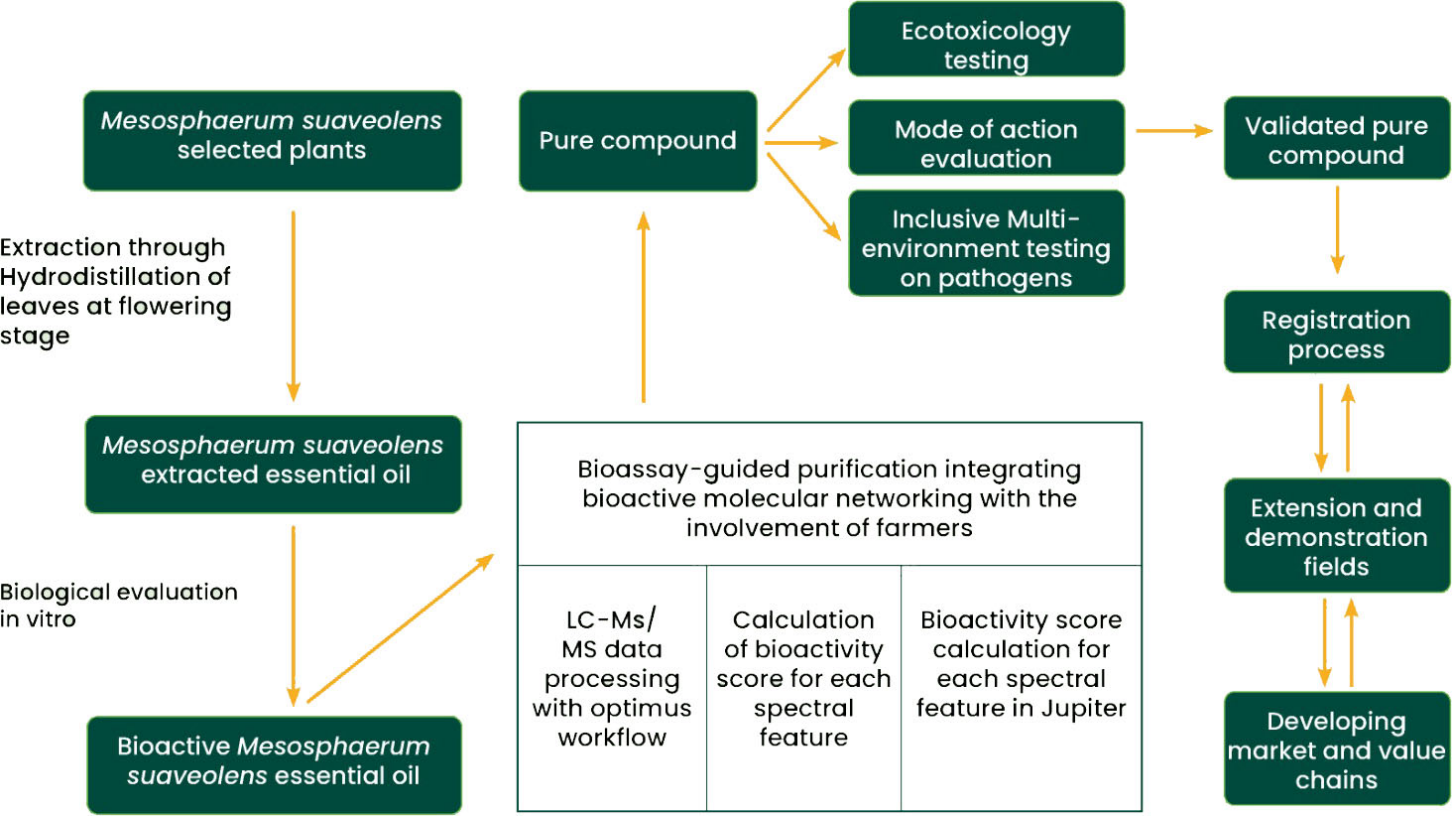
Process for *Mesosphaerum suaveolens* biopesticide development and scaling up.

5- Assessing the biological activity of the isolated constituents responsible for the insecticidal, fungicidal, nematicidal, and allelopathic effects of the volatile oil of *M. suaveolens*. Their synergistic effect should also be assessed. For the majority of *M. suaveolens* toxicity assessments, after using the whole extract, very few screenings have isolated and identified the potent phytotoxic substances of *M. suaveolens* and evaluated their potential toxicity on pests. There is an opportunity to increase the discovery of potent phytotoxic substances and also to follow and improve the existing discovery process of new potential biopesticides promoted by Pino et al. (2013) by adding multi-environmental testing, mode of action, toxicology, ecotoxicology, quality control, and stability studies aspects (**Figure 3**). While the bioactivity of the plant extract is demonstrated, a bioassay-guided fractionation should be carried out and commonly encompasses the five major steps: (i) extraction of metabolites from the plant materials, (ii) fractionation of the deriving extract by chromatography, (iii) bioassay assessment of each fraction, (iv) isolation of all molecules from the bioactive fractions, and (v) identification and evaluation of the bioactivity of the isolated molecules (Nothias et al., 2018).

6- Exploring the development of novel *M. suaveolens* essential oil derivatives for a durable pesticidal effect when applied in field conditions (Devrnja et al., 2022). Recent studies highlighted the importance of the application of new technologies for EOs-Based Insecticide (Pinto et al., 2022). Therefore, different nanocarriers have been used with success for the encapsulation of essential oils. The most used nanocarriers in food and agriculture include lipid nanoparticles, emulsions, clay-based nanoparticles, biopolymeric nanoparticles, and inclusion complexes (Kumar et al., 2022).

7- Exploring the development of high-yielding and stable varieties of *M. suaveolens*. The diversity aspects will help overcome the difficulties in field tests which require a sufficient quantity of essential oil. The development of high yielding still requires extensive investigations such as evaluation and conservation of genetic resources, development of molecular markers, best agronomic practices of production, essential oil extraction methods, assessment of biotic and abiotic factors, pollination, and hybrid production studies.

8- Elucidating the mode of action of *M. suaveolens* essential oil and its bioactive constituents on insects, viruses, fungi, and plants. Very few studies have been conducted on the mechanism of action of *M. suaveolens* and its main components, monoterpenes. *M. suaveolens* essential oil is generally applied as contact liquid, fumigants, or mixed with various solid ingredients (kaolin powder, and mycorrhizae). For example, the essential oil of *M. suaveolens* induced several mechanisms involving visible damage, reduction in chlorophyll content, and cell viability, leading to total wilting of the plants (Sharma et al., 2019). Investing in the discovery of the mode of action of the bioactive constituents of *M. suaveolens* could be a major step towards accelerating pesticide research and development, as it could reduce the time and cost required (Pino et al., 2013).

## 10 Major challenges in the use of *Mesosphaerum suaveolens* essential oil

The efficacy of *M. suaveolens* essential oil has been widely demonstrated on many pathogens throughout the world. With variable chemical content of essential oil extracted from different plant part, it has been widely recognized by many researchers as a potential biopesticide to be used in agriculture. The effectiveness of *M. suaveolens* essential oil has been reported against cereal weevils (maize, rice, etc.), leguminous pests (cowpea, groundnut, etc.), and vegetable pathogens (tomato, cabbage, etc.). Many research activities on the pesticidal properties of the oil have started more than two decades ago. However, like many other biopesticides based on plant essential oils, *M. suaveolens* products are not yet on the market. Several steps, including validation of laboratory results in the field, remain to be undertaken to reach the level of existing regulations and commercialization as bioinsecticides containing the two most common, azadirachtin and pyrethrin, found on the global markets. In addition to limiting factors inherent in the essential oil, such as variability in composition, volatility and poor persistence after application, external challenges related to farmer perceptions, investment in research into products derived from *M. suaveolens* essential oil, and regulations and approvals to facilitate commercialization need to be overcome. Despite the traditional use of *M. suaveolens* and knowledge of its repellent effect, its use by farmers for pest control is very limited. The reasons given for the low use of biopesticides are the time required to prepare the extracts, which is considered too long, and the number of treatments recommended, which is too high (Yarou et al., 2017) in Africa. Thus, the need for companies to invest would be almost non-existent, despite the global need for biopesticides to ensure food security and environmental protection in the current context of climate change. This suggests a global approach that includes the awareness of producers and agribusinesses, which would facilitate the implementation of research projects on products derived from *M. suaveolens* essential oils. Furthermore, according to Pino et al. (2013), the commercialization within the regulatory framework for plant-derived pesticide products, requires validation of the main barriers. These are the accessibility and sustainability of the genetic materials, the stability of the extracts, standardization of the active constituent, and regulatory permission. Research on the essential oil of *M. suaveolens* must include these criteria to facilitate the rapid marketing of pesticides derived from *M. suaveolens.* Wide dissemination of results and open dialogue with farmers will also be needed to facilitate the discovery process and finally the adoption of essential oil uses at large scale in the field.

## 11 Conclusion

Researchers have an increasing interest in the use of *M. suaveolens* in agriculture because of the bioactive chemical constituents found in its essential oil. However, considering the need for sustainable agriculture, extensive research remains to be carried out to overcome the existing limitations related to *M. suaveolens* essential oil. To date, most of the research studies have focused on the pesticidal and allelochemical properties of the essential oil of *M. suaveolens* in laboratory or greenhouse settings. Consequently, the validation of laboratory and greenhouse trials in field conditions is important for understanding and assessing the environmental impacts on the biological activities of *M. suaveolens* essential oil. Furthermore, more attention should be given to the toxicological effect of *M. suaveolens* EOs on beneficial organisms and plants. Through this review, we present the current state of allelopathy and pesticidal research and highlight the pesticidal and allelopathic potential in *M. suaveolens* essential oil as a source of eco-friendly pesticides and herbicides, while also identifying knowledge gaps that require further exploration for the introduction of natural pesticidal and allelopathic substances in agriculture.
